# Supplementary material for: Peroxisome Proliferator Activator α Agonist Clofibrate Induces Pexophagy in Coconut Oil-Based High-Fat Diet-Fed Rats
Source: Biology (Basel). 2024 Dec 7;13(12):1027. doi: 10.3390/biology13121027 (PMC11673738; doi:10.3390/biology13121027)
Supplement: Supplementary file 1 [file biology-13-01027-s001.zip › biology-3086473-supplementary.pdf]

Supplementary Materials

Peroxisome proliferator activator α induces pexophagy in coconut oil-based high fat diet-fed rats.

Kanami Ohshima[1], Emika Hara[1], Mio Takimoto [1], Yidan Bai [1], Mai Hirata [1], Wen Zhen[1], Suzuka Uomoto[1], Mai Todoroki [1,2], Mio Kobayashi[1,2], Takuma Kozono [3], Tetsuhito Kigata [4], Makoto Shibutani[1], Toshinori Yoshida[1*]

[1] Laboratory of Veterinary Pathology, Cooperative Department of Veterinary Medicine, Tokyo University of Agriculture and Technology, 3-5-8 Saiwai-cho, Fuchu-shi, Tokyo 183-8509, Japan

[2] Cooperative Division of Veterinary Sciences, Tokyo University of Agriculture and Technology, 3-5-8 Saiwai-cho, Fuchu-shi, Tokyo 183-8509, Japan

[3] Smart-Core-Facility Promotion Organization, 3-5-8 Saiwai-cho, Fuchu-shi, Tokyo 183-8509, Japan

[4] Laboratory of Veterinary Anatomy, Cooperative Department of Veterinary Medicine, Tokyo University of Agriculture and Technology, 3-5-8 Saiwai-cho, Fuchu-shi, Tokyo 183-8509, Japan

*Corresponding author: Toshinori Yoshida, D.V.M., Ph.D., Laboratory of Veterinary Pathology, Tokyo University of Agriculture and Technology, 3-5-8 Saiwai-cho, Fuchu-shi, Tokyo 183-8509, Japan. E-mail: yoshida7@cc.tuat.ac.jp; Tel +81-42-367-5874; Fax: +81-42-367-5771

Supplemental methods

Animal Experiment

We made a study plan on the base of a medium-term liver assay for 8 weeks to detect hepatocarcinogen for screening [27]. The research groups examined strain, age, sex, initiation substance, timing of partial hepatectomy for enhancing preneoplastic lesions, and specific markers of preneoplastic foci to well and specifically detect hepatocarcinogenesis of test substances [27,44]. According to these data, male F344 rats were sensitive to the female and other strains. In patients with NAFLD, males have a higher risk of steatosis, NASH with expression of pro-inflammatory and pro-fibrotic cytokines, and HCC than females [45]. So we selected male rats in our study. This model allows us to evaluate precancerous lesions enhanced by fatty liver-related NADPH oxidase [28,29], and autophagy [30-32].

Histopathology

The fixed liver slices were routinely dehydrated in graded ethanol, embedded in paraffin, sectioned, and stained with hematoxylin and eosin for histopathological examination. A histopathological examination was conducted using microscopy (BX53; Olympus Corporation, Hachioji, Tokyo, Japan) with magnification, x40, x100, x200, and x400. The hepatic pathological changes including steatosis, ballooning change, and inflammatory foci were graded by the NAFLD activity score (NAS) [29,36,37]; The H&E-stained specimens were evaluated by scoring the extent of celluar changes based on steatosis (0, <5%; 1, 5%–33%; 2, 33%–66%; and 3, >66%), ballooning change (0, none; 1, few balloon cells; and 2, many), and inflammatory foci (0, no foci; 1, <2 foci; 2, 2-4 foci; and 3, >4 foci).

Ultrastructural Examination

Fresh liver samples were cut into 1 mm squares and re-fixed in 2.5% glutaraldehyde solution for 24 hours. The samples were washed with 0.01M phosphate buffer and stored at -4°C. They were postfixed with 1% osmium tetroxide (Nisshin EM, #300, Tokyo, Japan) for an hour. The samples were then permeabilized stepwise in 50% to 100% ethanol for lipophilic treatment and were shaken with propylene oxide (Nisshin EM, #311, Tokyo, Japan). The samples were then embedded in epoxy resin (TAAB, #T024, Aldermaston, UK). The semi-thin sections were prepared with Ultramicrotome EM UC7 (Leica, Wetzlar, Germany), and stained with toluidine blue (Muto Pure Chemicals Co., Ltd. #10331, Tokyo, Japan) to select centrilobular and mid-lobular regions of hepatic lobules. Ultra-sectioned at 80 nm were stained with EM stainer (Nisshin EM, #311, Tokyo, Japan) and lead to observe with a transmission electron microscope (TEM), JEM-1400Flash (JEOL Ltd., Tokyo, Japan) with magnification, x20 to x40k.

Immunohistochemistry

Paraffin-embedded liver sections were deparaffinized as described above. The deparaffinized liver sections were treated with 0.3% H2O2 in methanol for 30 min, and then antigen retrieval was performed. The sections were incubated with primary antibodies overnight at 4°C. Antigen retrieval and dilution of primary antibodies were conducted according to Supplemental Table 1. An avidin–biotin–peroxidase complex method with VECTASTAINR Elite ABC Kit (Vector Laboratories Inc., Burlingame, CA) was then employed with 3,3'-diaminobenzidine as a chromogen. The sections with positive reactions were counterstained with hematoxylin. For GST-P-positive precancerous lesions, the number and area of lesions > 0.1 mm in diameter and total liver area per were calculated using Fiji (Image J, https://imagej.net/software/fiji/downloads) [27]. Granules that expressed Pex5, NBR1, ADRP, LAMP2, and p62 in hepatocytes were measured for positivity rate per unit area (a total of three areas at x400) using Fiji. An immunohistochemical examination was conducted using microscopy (BX53, Olympus Corporation) with a magnification of x400 for Pex5, NBR1, ADRP, LAMP2, and p62, and a virtual slide system (VS120, Olympus Corporation) for GST-P.

Real-Time Reverse Transcription-Polymerase Chain Reaction Analysis

Total RNA was extracted with RNeasyR Mini Kits (Qiagen, Hilden, Germany) according to the manufacturer's instructions, as previously reported [28]. First-strand cDNA was synthesized from 2 μg total RNA in a 20 μl total reaction mixture containing dithiothreitol, deoxynucleoside triphosphates, random primers, RNaseOUT, and SuperScript™III Reverse Transcriptase (Life Technologies, Carlsbad, CA). Real-time RT-PCR was performed using the SYBRR Green PCR Master Mix (Life Technologies) and the StepOnePlus™ Real-Time PCR System (Life Technologies) according to the manufacturer's protocol. The PCR primers were designed using Primer Express software (version 3.0; Life Technologies). The relative differences in gene expression were calculated using threshold cycle (CT) values that were first normalized to those of the hypoxanthine phosphoribosyl transferase 1 gene, the endogenous control in the same sample, and then relative to a control CT value by the $2^{—\Delta\Delta C}$ T method [39].

Table S1. Antibodies for immunohistochemistry

Antigen	Host species	Clonality (Clone)	Dilution	Antigen retrieval	Vender
ADRP	Mouse	Monoclonal	1:1000	Microwaving at 90°C for 10 min in citrate buffer, pH6.0	Proteintech Group, Inc (IL, USA)
GST-P	Rabbit	Polyclonal	1:1000	None	Medical & Biological Laboratories (Nagoya, Japan)
LAMP2	Rabbit	Polyclonal	1:500	Microwaving at 90°C for 10 min in citrate buffer, pH9.0	Bioss (MA, USA)
NBR1	Rabbit	Polyclonal	1:1200	Microwaving at 90°C for 10 min in citrate buffer, pH9.0	Proteintech Group, Inc (IL, USA)
p62; SQSTM1	Rabbit	Polyclonal	1:500	Microwaving at 90°C for 10 min in citrate buffer, pH9.0	Proteintech Group, Inc (IL, USA)
Pex5	Rabbit	Polyclonal	1:800	Microwaving at 90°C for 10 min in citrate buffer, pH9.0	Proteintech Group, Inc (IL, USA)

Abbreviations: ADRP, Adipophilin; GST-P, Placental glutathione S-transferase; LAMP2, Lysosomal associated membrane protein 2; NBR1, Neighbor of BRCA1 gene 1; Pex5, Peroxisomal biogenesis factor 5.

Table S2. Sequence of primers used for real-time RT-PCR

Accession no.	Gene	Forward primer (5`→3`)	Reverse primer (5`→3`)
NM 178095.3	*Abca1*	CCAGGAGCGTGTGAGCAAAG	ACCAGTGTAGCAGGGACCACATAA
NM 017340	*Acox1*	GCGCAAGGAGCGGGCCTCC	CTCGACGGCGCCGGGTATTC
NM_145770.3	*Acox2*	TGGAGCGACTCACCAACATC	GCTCCTCTCGGGTCATGAAG
NM 019287.2	*Apob*	TGCGGTGGCAGAAATAACG	AGTAGCCAGAGAGCTGGTCTGA
NM 134394.2	*Atg3*	GCAGCACCATGCAGGTGAG	TGGTCACTCGGTCCAGGATC
NM 001014250.1	*Atg5*	CTGTTCGATCTTCTTGCATCA	TCCTTTTCTGGAAAACTCTTGAA
NM 001012097.1	*Atg7*	TTCTTAGAAGATTTGACTGGTCTTACA	TCACTCATGTCCCAGATCTCA
NM 012520	*Catalase*	ATTGCCGTCCGATTCTCC	CCAGTTACCATCTTCAGTGTAG
NM 017332	*Fasn*	GCGGGCGTGGTAATGCT	CTGTTCGCAAATACGCTCCAT
NM 030826	*Gpx1*	GCTGCTCATTGAGAATGTCG	GAATCTCTTCATTCTTGCCATT
NM 012857	*Lamp1*	GCAAGGCGCTCGCCCTCAAT	GCCCGCGTGACTCCTCTTCC
NM 017068	*Lamp2*	AGCAGGTGGTTTCCGTGTCTCG	AGGGCTGCTCCCACCGCTAT
NM 022867	*Lc3*	CGGGTTGAGGAGACACACAA	TCTTTGTTCGAAGCTCCGGC
AF227191.1	*Nbr1*	ATGGAACCACAGGTTACTCTAAA	CTGGATCCGAAACCAGAAAGC
NM 175843.4	*P62*	CGGAAGTCAGCAAACC	ATGCGTCCAGTCGTCA
NM_172063.1	*Pex14*	CTCGAGAGCCTCTGATTGCC	AAATGCTCTCCTGGTCGCAA
NM_017234.2	*Pex2*	GTGGCCCACAATGCCTCATA	ACTTCTGTGCCACACTTAGGG
NM_001170584.1	*Pex5*	GGAACCTCTACCACTGACCG	ATGTGCCCTCAGAGTTAGCC
NM 013196	*Ppara*	CCCCACTTGAAGCAGATGACC	CCCTAAGTACTGGTAGTCCGC
NM 001145366	*Pparg*	GACCACTCCCATTCCTTTGA	CATTGGGTCAGCTTGTGA
NM 139192	*Scd1*	CACACGCCGACCCTCACAACT	TCCGCCCTTCTCTTTGACAGCC
NM_017050.1	*Sod1*	CATTCCATCATTGGCCGTACTA	TTTCCACCTTTGCCCAAGTC
NM 017051.2	*Sod2*	CTCCCTGACCTGCCTTACGA	CTGCATGATCTGCGCGTTA
NM_012880.2	*Sod3*	CCTCCTTCAATCTGGAGGGC	GGTTGTAGTGTGGTCCGGTG
NM 001276707.1	*Srebf1*	GGAGCCATGGATTGCACATT	GCTTCCAGAGAGGAGCCCAG

Table S3. Body weight change in rats

Group	CTL	CF	HFD	HFD+CF
No. of animals	5	6	6	7
1‡	126.8±12.0[a]	116.4±4.7[b]	110.3±3.1[b]	107.2±5.0[b]
2	147.4±9.3[a]	146.9±6.1[a]	134.3±3.3[b]	128.4±6.5[b]
3	182.9±8.6[a]	182.8±6.7[a]	167.1±3.7[b]	161.3±7.3[b]
4	197.6±5.9[a]	193.3±7.2[ab]	185.7±3.8[bc]	176.0±8.4[c]
5	217.0±5.8[a]	200.4±12.5[ab]	203.1±3.0[a]	185.8±11.6[b]
6	231.8±8.2[a]	218.5±10.9[a]	222.1±4.4[a]	200.6±10.4[b]
7	244.5±9.0[a]	229.6±10.2[ab]	235.6±5.8[ab]	218.5±18.0[b]
8	255.9±8.6[a]	240.7±10.6[ab]	251.7±6.6[a]	229.1±10.2[b]
9	262.7±8.0[a]	248.6±10.4[ab]	261.1±6.1[a]	239.0±11.1[b]
10	271.6±8.4[a]	255.4±10.2[b]	273.0±6.2[a]	246.7±11.1[b]
11	272.4±9.2[ab]	258.4±10.5[a]	282.8±5.6[b]	253.9±10.7[a]
12	281.6±11.8[a]	264.1±11.8[b]	289.4±5.3[a]	259.9±11.0[b]
13	291.6±10.1[a]	267.3±10.7[b]	293.9±5.2[a]	265.8±10.8[b]
14	295.4±11.1[a]	271.1±10.8[b]	295.5±5.7[a]	267.7±11.0[b]

Abbreviations: BW, body weight; CF, clofibrate; CTL, control diet; HFD, high-fat diet.

‡: Study week.

Data are shown as the mean±standard deviation (g).

Different letters indicate significant differences between groups ($p<0.05$, significantly different by Tukey's or Steel-Dwass test).

Table S4. Food intake in rats

Group	CTL	CF	HFD	HFD+CF
No. of animals	5	6	6	7
1‡	12.50	12.46	8.41	8.27
2	13.94	13.94	9.02	8.85
3	12.79	13.85	8.87	8.28
4	9.65	8.78	8.28	5.88
5	13.43	12.19	9.55	11.65
6	12.64	13.61	8.98	12.78
7	11.59	12.27	8.11	13.82
8	12.17	13.23	8.60	10.68
9	11.64	10.95	8.32	10.33
10	12.32	11.82	10.91	9.48
11	9.45	12.66	8.08	9.05
12	14.73	11.19	7.00	8.90
13	11.67	10.41	7.72	9.37
14	11.15	12.89	7.01	8.74

Abbreviations: CF, clofibrate; CTL, control diet; HFD, high-fat diet.

‡: Study week.

Data are shown as the mean (g/rat/day).

Table S5. Water intake in rats

Group	CTL	CF	HFD	HFD+CF
No. of animals	5	6	6	7
1‡	11.60	10.64	8.74	13.35
2	19.60	18.74	17.96	19.65
3	18.64	17.76	19.48	19.23
4	14.78	13.87	16.00	12.97
5	17.47	17.43	19.86	17.83
6	16.00	16.71	17.02	16.61
7	15.92	16.50	17.02	19.94
8	15.79	16.61	16.82	17.74
9	16.26	15.52	10.96	16.74
10	15.15	14.40	14.29	15.01
11	12.28	14.61	13.63	13.99
12	16.03	14.00	12.41	13.82
13	14.11	13.71	12.42	14.75
14	14.75	14.00	14.85	13.43

Abbreviations: CF, clofibrate; CTL, control diet; HFD, high-fat diet.

‡: Study week.

Data are shown as the mean (g/rat/day).

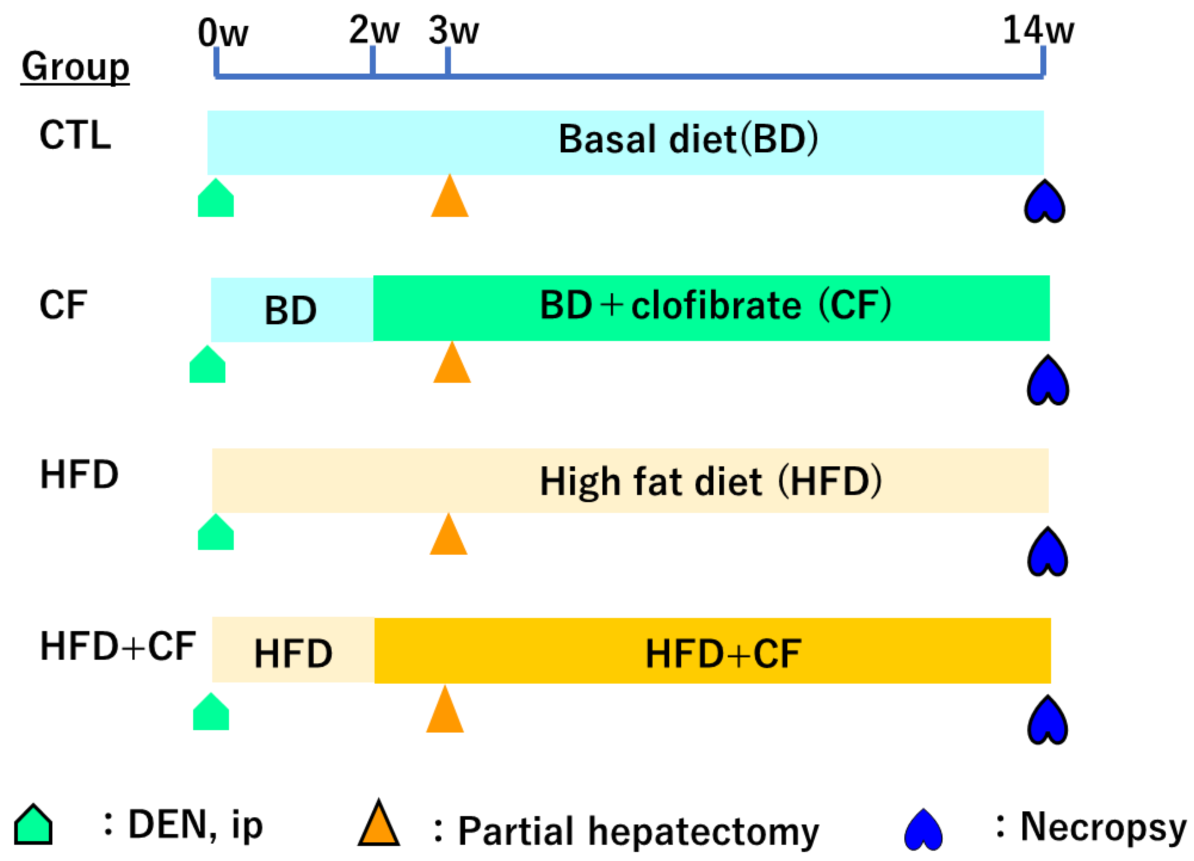

Figure S1. Study design.

Male F344/DuCrlCrlj rats were subdivided into four groups: a basal diet-fed group (CTL group, six rats), basal diet-fed and CF-administered group (CF group, six rats), HFD containing coconut oil-fed group (HFD group, six rats), and combined HFD and CF administration group (HFD+CF group, seven rats). According to established two-step (initiation and promotion) hepatic carcinogenesis model, all rats was taken by intraperitoneal administration of DEN (200 mg/kg body weight) at week 0 (initiation phase), and subjected 2/3 partial hepatectomy under deep isoflurane anesthesia at week 3. Rapid cell proliferation is a prerequisite for effective initiation by a single treatment with DEN, a hepatocarcinogen as well as being important during the promotion phase. Rats received each test diet from week 2 to week 14 (promotion phase). At 14 weeks after DEN administration, after overnight fasting (water supply continued) , all animals were subjected to necropsy.

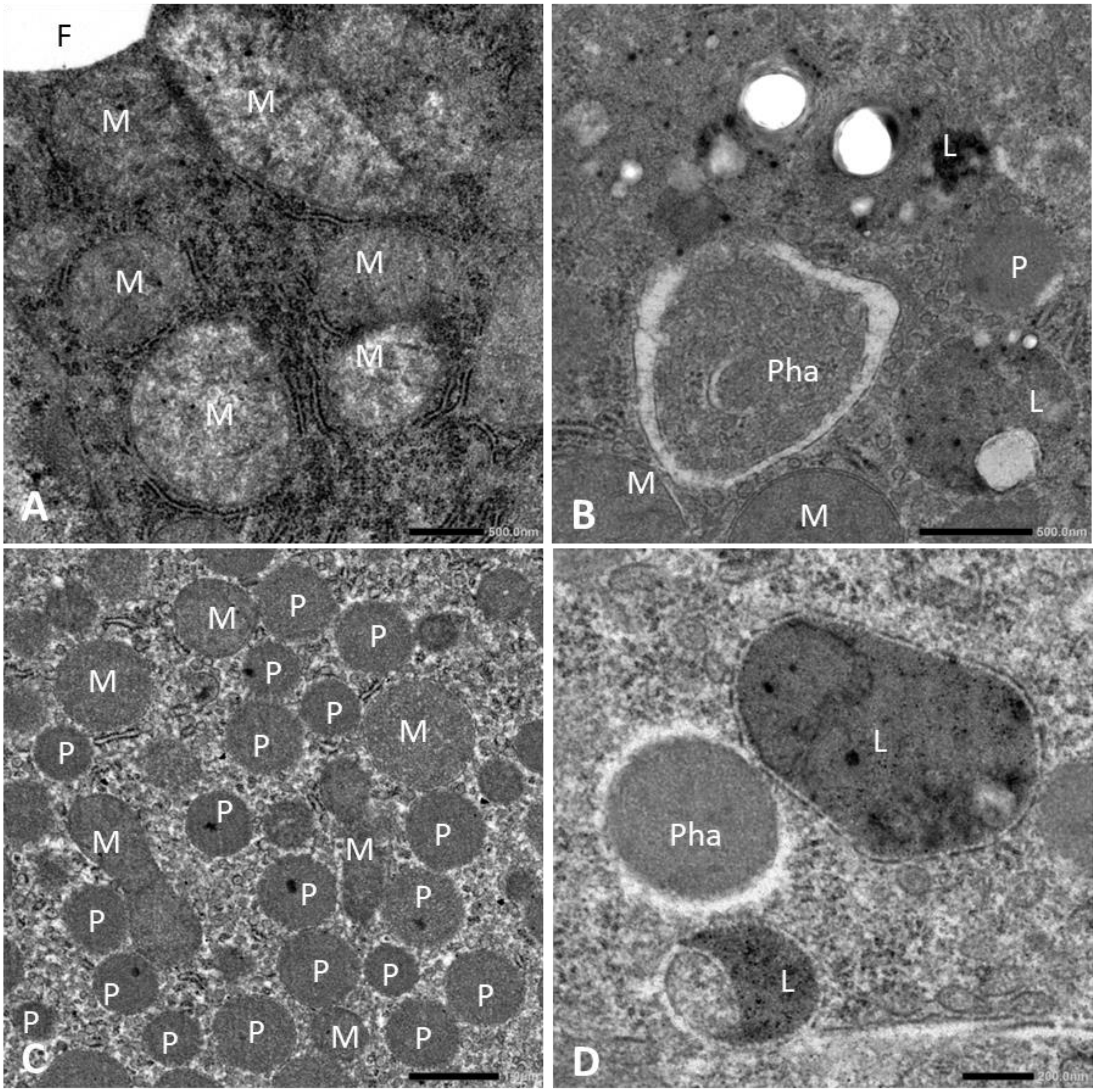

Figure S2. Representative TEM images of peroxisome and phagophores of hepatocytes.

(A) Enlargement of mitochondria (M) and fat droplet (F) in the HFD group. B) Peroxisomes (P), mitochondria, phagophore (Pha), autolysosomes (L) in the CF group. (C, D) Peroxisomes, mitochondria, phagophores, and autolysosomes in the HFD+CF group. Numerous peroxisomes are observed, along with mitochondria. In a phagophore, an organelle, possibly peroxisome is captured by phagophore, indicating phagophore expansion (B, D). TEM, transmission electron microscopy. Bar=200 (D), 500 nm (A, B) and 1.0 µm (C).

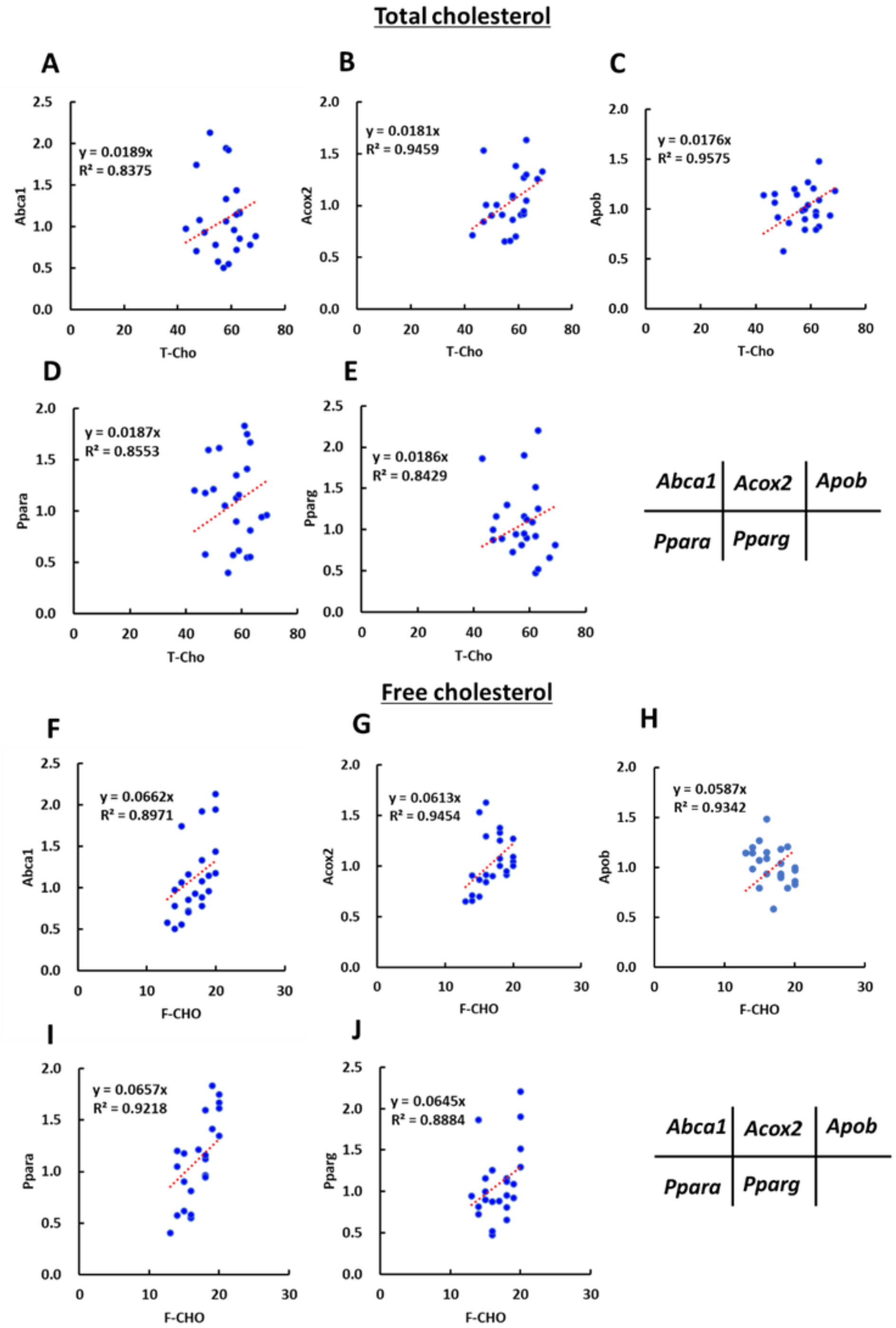

Figure S3. Correlation plots of plasma cholesterol with expression levels of lipid metabolism-related genes. (A-E) Comparison of T-CHO in the plasma with gene expression levels of *Abca1*, *Acox2*, *Apob*, *Ppara*, and *Pparg* in the liver. (F-J) Comparison of F-CHO in the plasma with gene expression levels of *Abca1*, *Acox2*, *Apob*, *Ppara*, and *Pparg* in the liver. Y=ax and R^2 are shown in each comparison. T-CHO, total cholesterol; F-CHO, free cholesterol.

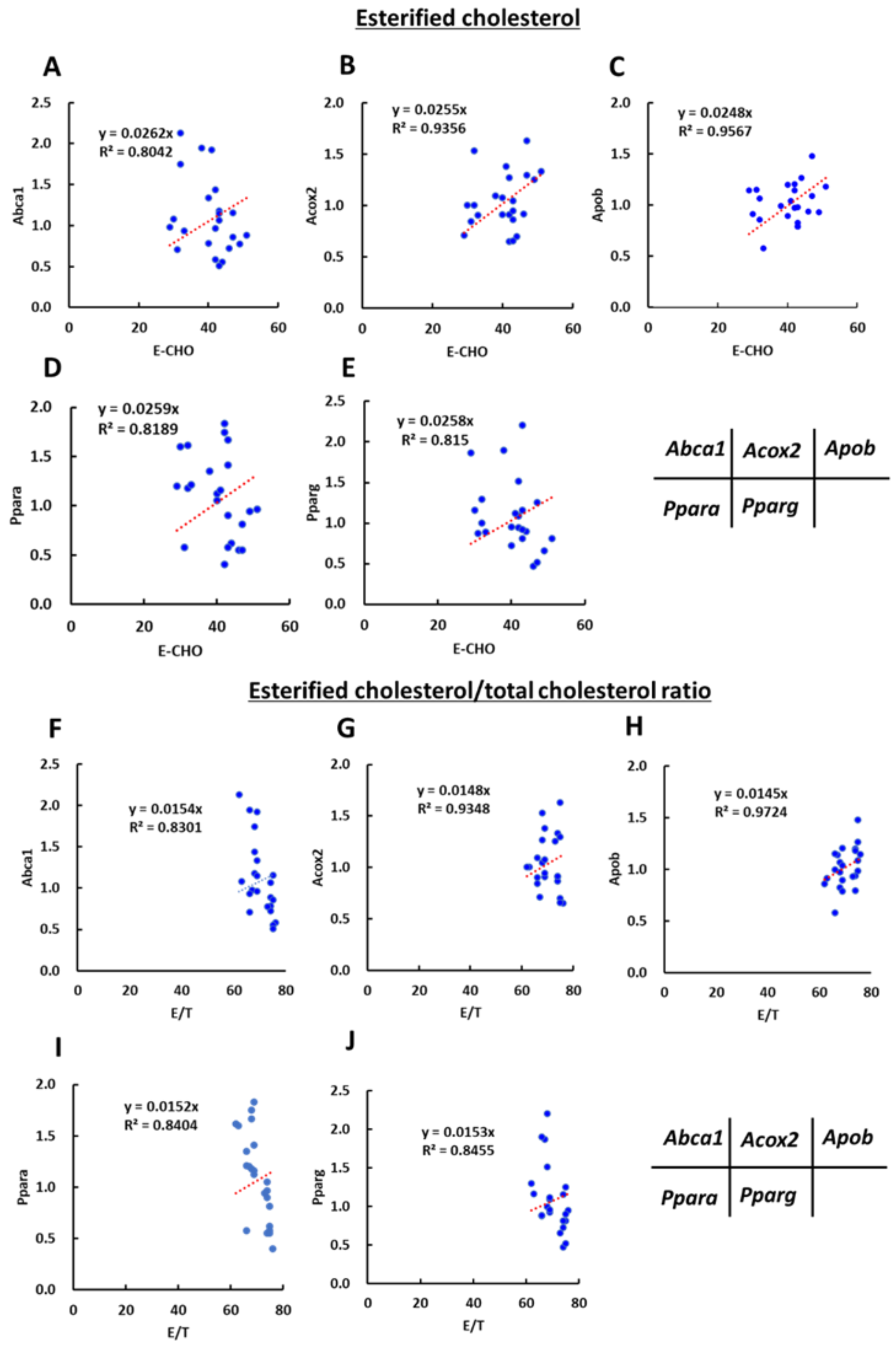

Figure S4. Correlation plots of plasma esterified cholesterol with expression levels of lipid metabolism-related genes. (A-E) Comparison of E-CHO in the plasma with gene expression levels of *Abca1*, *Acox2*, *Apob*, *Ppara*, and *Pparg* in the liver. (F-J) Comparison of E/T ratio with gene expression levels of *Abca1*, *Acox2*, *Apob*, *Ppara*, and *Pparg* in the liver. Y=ax and R^2 are shown in each comparison. E-CHO, esterified cholesterol; E/T, esterified cholesterol/total cholesterol ratio.

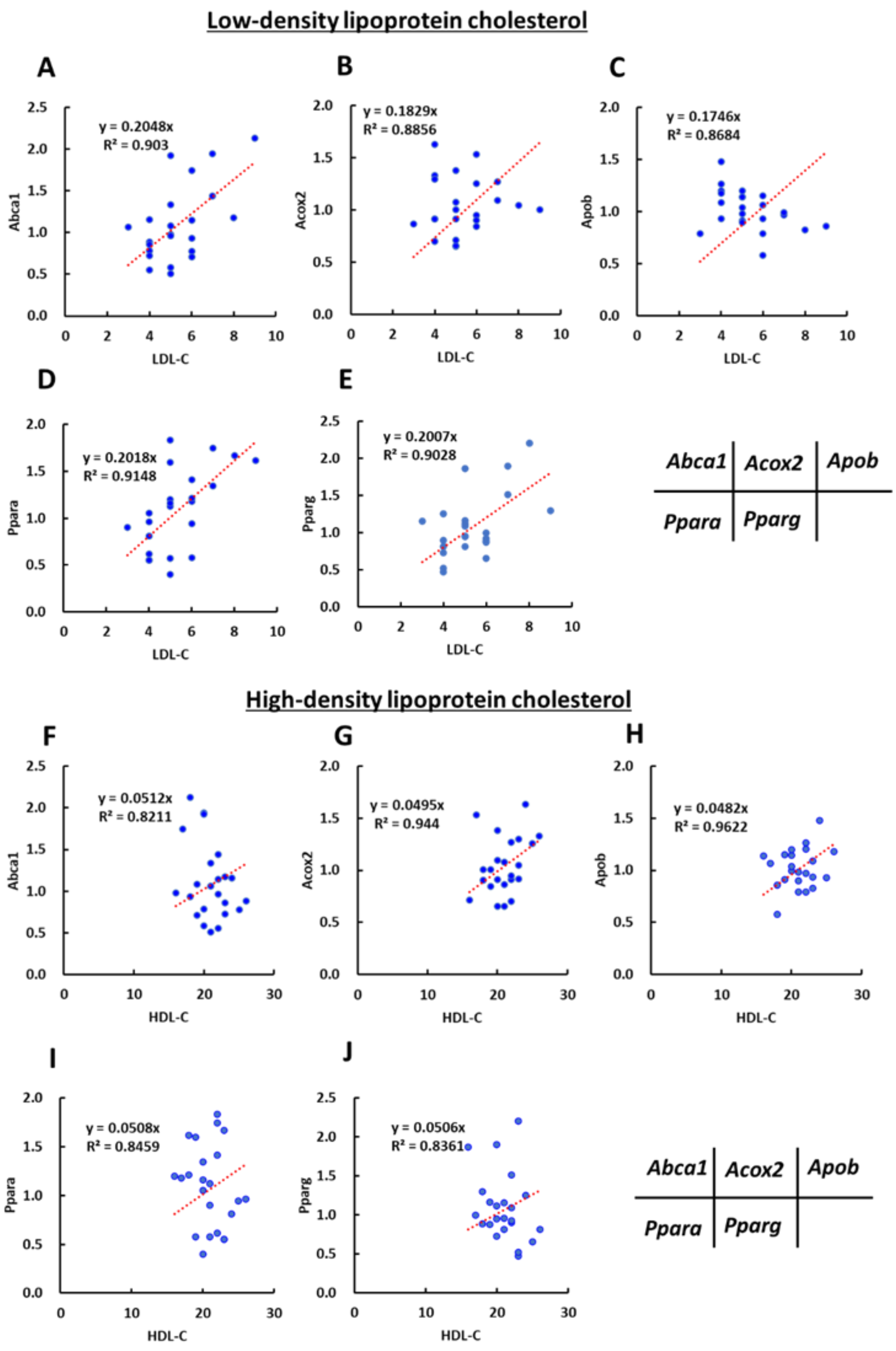

Figure S5. Correlation plots of plasma lipoprotein cholesterol with expression levels of lipid metabolism-related genes. (A-E) Comparison of LDL-C in the plasma with gene expression levels of *Abca1*, *Acox2*, *Apob*, *Ppara*, and *Pparg* in the liver. (F-J) Comparison of HDL-C with gene expression levels of *Abca1*, *Acox2*, *Apob*, *Ppara*, and *Pparg* in the liver. Y=ax and R^2 are shown in each comparison. LDL-C, low-density lipoprotein cholesterol; HDL-C, high-density lipoprotein cholesterol.

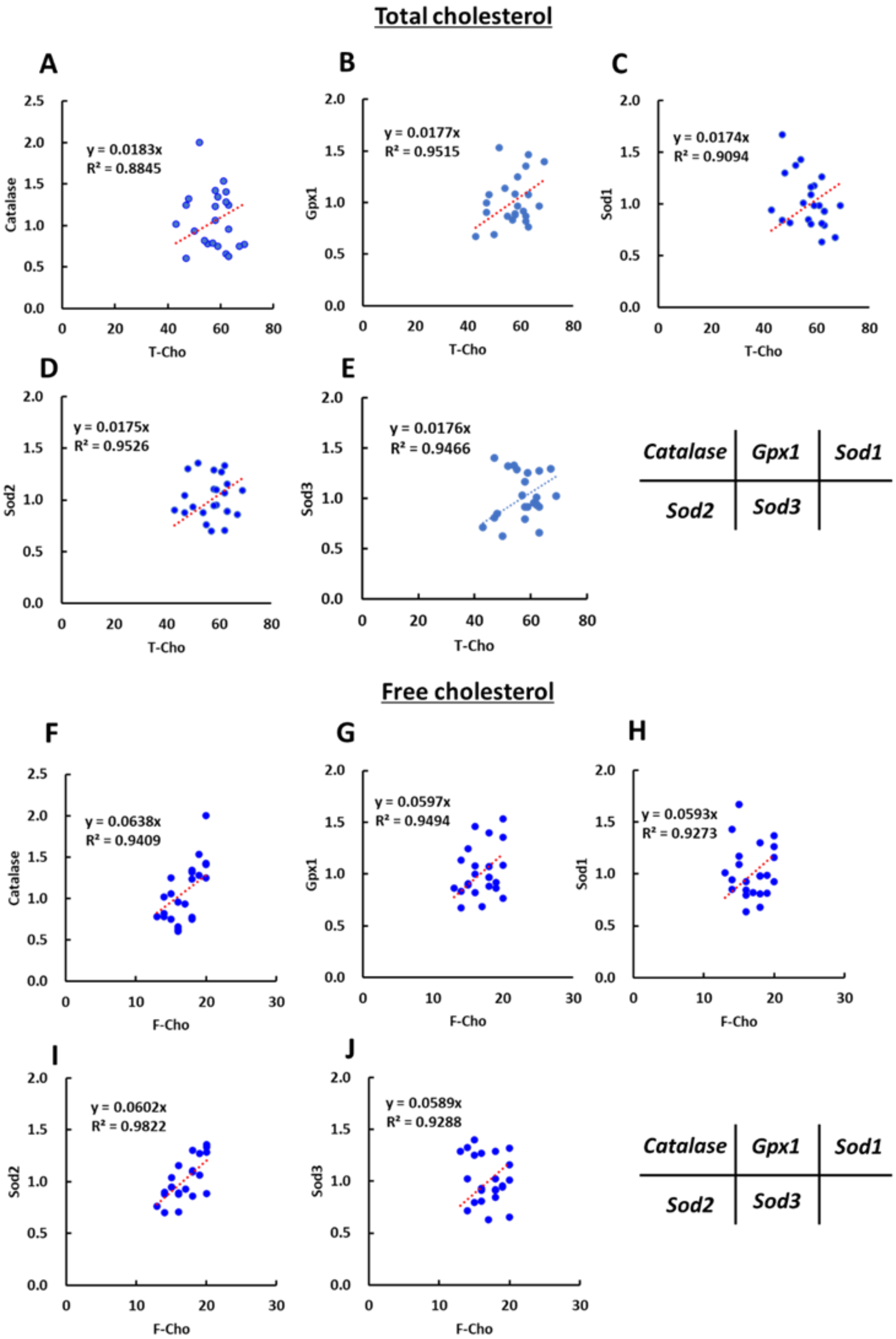

Figure S6. Correlation plots of plasma cholesterol with expression levels of antioxidant response genes. (A-E) Comparison of T-CHO in the plasma with gene expression levels of *Catalase, Gpx1, Sod1, Sod2,* and *Sod3* in the liver. (F-J) Comparison of F-CHO in the plasma with gene expression levels of *Catalase, Gpx1, Sod1, Sod2,* and *Sod3* in the liver. Y=ax and R^2 are shown in each comparison. T-CHO, total cholesterol; F-CHO, free cholesterol.

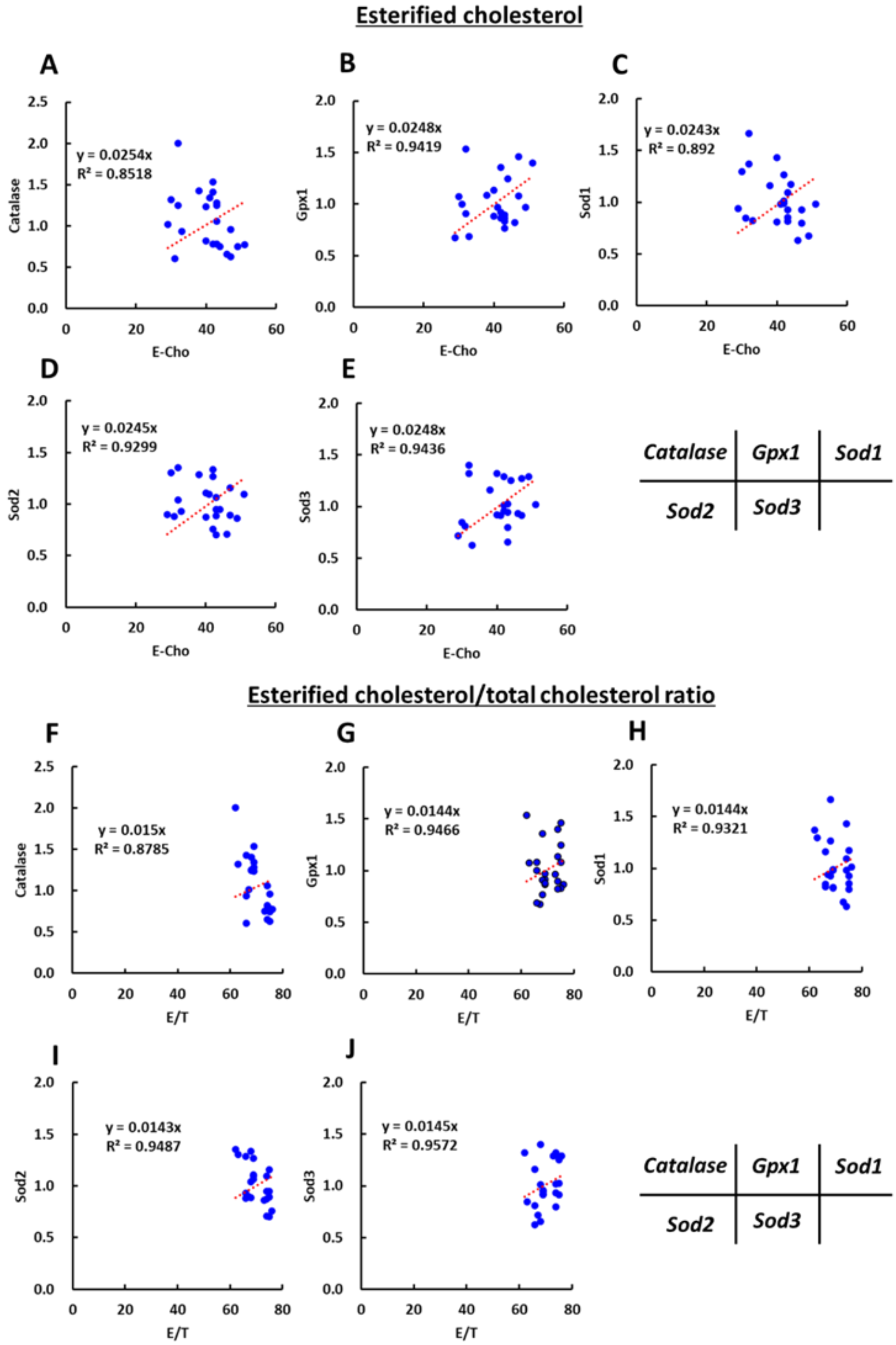

Figure S7. Correlation plots of plasma esterified cholesterol with expression levels of antioxidant response genes. (A-E) Comparison of E-CHO in the plasma with gene expression levels of *Catalase, Gpx1*, *Sod1*, *Sod2*, and *Sod3* in the liver. (F-J) Comparison of E/T ratio with gene expression levels of *Catalase, Gpx1*, *Sod1*, *Sod2*, and *Sod3* in the liver. Y=ax and R^2 are shown in each comparison. E-CHO, esterified cholesterol; E/T, esterified cholesterol/total cholesterol ratio.

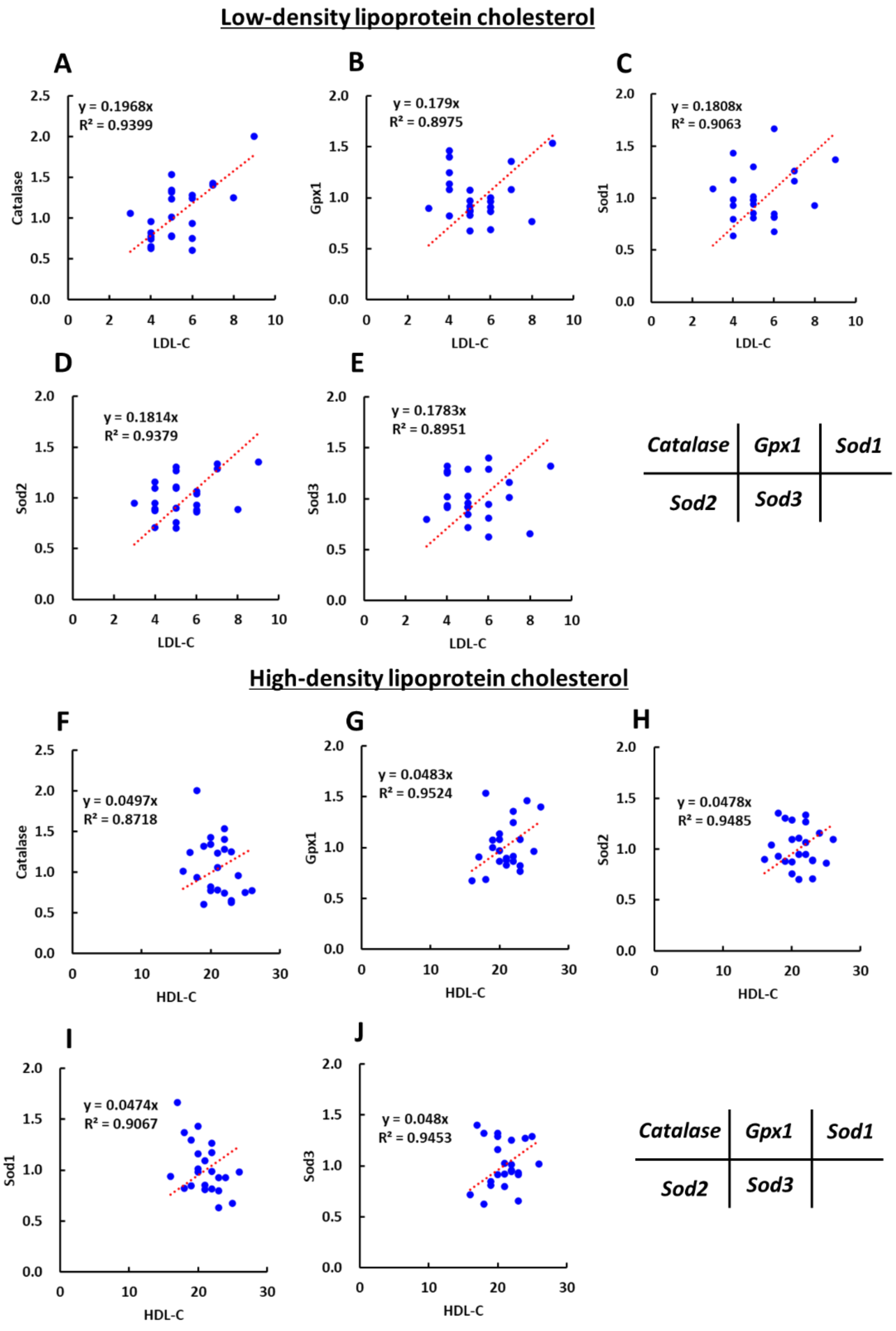

Figure S8. Correlation plots of plasma lipoprotein cholesterol with expression levels of antioxidant response genes. (A-E) Comparison of LDL-C in the plasma with gene expression levels of *Catalase, Gpx1, Sod1, Sod2,* and *Sod3* in the liver. (F-J) Comparison of HDL-C with gene expression levels of *Catalase*, *Gpx1*, *Sod1*, *Sod2*, and *Sod3* in the liver. Y=ax and R^2 are shown in each comparison. LDL-C, low-density lipoprotein cholesterol; HDL-C, high-density lipoprotein cholesterol.
